# Delineating COVID-19 subgroups using routine clinical data identifies distinct in-hospital outcomes

**DOI:** 10.1038/s41598-023-32469-9

**Published:** 2023-06-20

**Authors:** Bojidar Rangelov, Alexandra Young, Watjana Lilaonitkul, Shahab Aslani, Paul Taylor, Eyjólfur Guðmundsson, Qianye Yang, Yipeng Hu, John R. Hurst, David J. Hawkes, Joseph Jacob, Тhe NCCID Collaborative, Pardeep Bains, Dominic Cushnan, Mark Halling-Brown, Joseph Jacob, Emily Jefferson, Francois Lemarchand, Anastasios Sarellas, Daniel Schofield, James Sutherland, Mathew Watt, Daniel Alexander, Hena Aziz, John R. Hurst, Emma Lewis, Gerald Lip, Peter Manser, Philip Quinlan, Neil Sebire, Andrew Swift, Smita Shetty, Peter Williams, Oscar Bennett, Samie Dorgham, Alberto Favaro, Samantha Gan, Tara Ganepola, Gergely Imreh, Neha Puri, Jonathan Carl Luis Rodrigues, Helen Oliver, Benjamin Hudson, Graham Robinson, Richard Wood, Annette Moreton, Katy Lomas, Nigel Marchbank, Chinnoi Law, Harmeet Chana, Nemi Gandy, Ban Sharif, Leila Ismail, Jaymini Patel, Debbie Wai, Liz Mathers, Rachel Clark, Anisha Harrar, Alison Bettany, Kieran Foley, Carla Pothecary, Stephen Buckle, Lisa Roche, Aarti Shah, Fiona Kirkham, Hannah Bown, Simon Seal, Hayley Connoley, Jenna Tugwell-Allsup, Bethan Wyn Owen, Mary Jones, Andrew Moth, Jordan Colman, Giles Maskell, Daniel Kim, Alexander Sanchez-Cabello, Hannah Lewis, Matthew Thorley, Ross Kruger, Madalina Chifu, Nicholas Ashley, Susanne Spas, Angela Bates, Peter Halson, Chris Heafey, Caroline McCann, David McCreavy, Dileep Duvva, Tze Siah, Janet Deane, Emily Pearlman, James MacKay, Melissa Sia, Esme Easter, Doreen Brookes, Paul Burford, Ramona-Rita Barbara, Thomas Payne, Mark Ingram, Bahadar Bhatia, Sarah Yusuf, Fiona Rotherham, Gayle Warren, Angela Heeney, Angela Bowen, Adele Wilson, Zahida Hussain, Joanne Kellett, Rachael Harrison, Janet Watkins, Lisa Patterson, Tom Welsh, Dawn Redwood, Natasha Greig, Lindsay Van Pelt, Susan Palmer, Kate Milne, Joanna Tilley, Melissa Alexander, Amy J. Frary, Judith L. Babar, Timothy Sadler, Edward Neil-Gallacher, Sarah Cardona, Avneet Gill, Nnenna Omeje, Claire Ridgeon, Fergus Gleeson, Annette Johnstone, Russell Frood, Mohammed Atif Rabani, Andrew Scarsbrook, Mark D. Lyttle, Stephen Lyen, Gareth James, Sarah Sheedy, Kiarna Homer, Alison Glover, Ben Gibbison, Jane Blazeby, Mai Baquedano, Thomas Payne, Teresa Jacob, Sisa Grubnic, Tony Crick, Debbie Crawford, Fiona Prestwood, Margaret Cooper, Mark Radon

**Affiliations:** 1 Satsuma Lab, Centre for Medical Image Computing (CMIC), University College London, London, UK; 2 Department of Neuroimaging, King’s College London, London, UK; 3 Institute of Health Informatics, University College London, London, UK; 4 Wellcome/EPSRC Centre for Interventional and Surgical Sciences, University College London, London, UK; 5 Centre for Medical Image Computing, University College London, London, UK; 6 UCL Respiratory, University College London, London, UK; 7 AI Lab, NHSX, Skipton House, 80 London Road, London, SE1 6LH UK; 8 Scientific Computing, Royal Surrey NHS Foundation Trust, Egerton Road, Guildford, GU2 7XX UK; 9 Health Data Research UK, Gibbs Building, 215 Euston Road, London, NW1 2BE UK; 10 Health Informatics Centre (HIC), School of Medicine, University of Dundee, Dundee, DD1 4HN UK; 11 Health Informatics Centre (HIC), School of Medicine, University of Dundee, (Main Level 5 Corridor), Second Floor, Level 7, Mailbox 15, Ninewells Hospital & Medical School, Dundee, DD1 9SY2 UK; 12 Department of Scientific Computing, Royal Surrey NHS Foundation Trust, Egerton Road, Guildford, GU2 7XX England; 13 Centre for Vision, Speech and Signal Processing, University of Surrey, Guildford, England; 14 North East Scotland Breast Screening Programme, NHS Grampian, Aberdeen, UK; 15 Digital Research Service, University of Nottingham, Nottingham, UK; 16 School of Medicine, University of Nottingham, Nottingham, UK; 17 Great Ormond Street Hospital, London, UK; 18 Department of Infection, Immunity and Cardiovascular Disease, University of Sheffield, Sheffield, S10 2RX UK; 19 Faculty, 54 Welbeck Street, London, W1G 9XS UK; 20 Royal United Hospitals Bath NHS Foundation Trust, Bath, UK; 21 Brighton and Sussex University Hospitals NHS Trust, Brighton, UK; 22 London North West University Healthcare NHS Trust, Harrow, UK; 23 George Eliot Hospital NHS Trust, Nuneaton, UK; 24 Cwm Taf Morgannwg University Health Board, Caerphilly, UK; 25 Hampshire Hospitals NHS Foundation Trust, Basingstoke, UK; 26 Betsi Cadwaladr University Health Board, Bangor, UK; 27 Ashford and St Peter’s Hospitals, Ashford, UK; 28 Royal Cornwall Hospitals NHS Trust, Truro, UK; 29 Sheffield Children’s NHS Foundation Trust, Sheffield, UK; 30 Liverpool Heart and Chest Hospital NHS Foundation Trust, Liverpool, UK; 31 Norfolk and Norwich University Hospitals NHS Foundation Trust, Norfolk, UK; 32 Royal Surrey NHS Foundation Trust, Guildford, UK; 33 Sandwell and West Birmingham NHS Trust, Birmingham, UK; 34 West Suffolk NHS Foundation Trust, Bury Saint Edmunds, UK; 35 Somerset NHS Foundation Trust, Taunton, UK; 36 Cambridge University Hospitals NHS Foundation Trust, Cambridge, UK; 37 Imperial College Healthcare NHS Trust, London, UK; 38 Oxford University Hospitals NHS Foundation Trust, Oxford, UK; 39 Leeds Teaching Hospitals NHS Trust, Leeds, UK; 40 University Hospitals Bristol NHS Foundation Trust, Bristol, UK; 41 St George’s Hospital NHS Foundation Trust, London, UK; 42 University Hospitals Of Morecambe Bay NHS Foundation Trust, Kendal, UK; 43 The Walton Centre, Liverpool, England

**Keywords:** Prognostic markers, Biomarkers, Infectious diseases, Scientific data, Statistics

## Abstract

The COVID-19 pandemic has been a great challenge to healthcare systems worldwide. It highlighted the need for robust predictive models which can be readily deployed to uncover heterogeneities in disease course, aid decision-making and prioritise treatment. We adapted an unsupervised data-driven model—SuStaIn, to be utilised for short-term infectious disease like COVID-19, based on 11 commonly recorded clinical measures. We used 1344 patients from the National COVID-19 Chest Imaging Database (NCCID), hospitalised for RT-PCR confirmed COVID-19 disease, splitting them equally into a training and an independent validation cohort. We discovered three COVID-19 subtypes (*General Haemodynamic*, *Renal* and *Immunological*) and introduced disease severity stages, both of which were predictive of distinct risks of in-hospital mortality or escalation of treatment, when analysed using Cox Proportional Hazards models. A low-risk Normal-appearing subtype was also discovered. The model and our full pipeline are available online and can be adapted for future outbreaks of COVID-19 or other infectious disease.

## Introduction

The COVID-19 pandemic, caused by the rapid spread of the original SARS-CoV-2 virus (and its follow-on variants) is one of the greatest health challenges faced in the modern age. As of May 2022 the global death toll exceeds 6.3 million people with more than 544 million confirmed infections^1^. Even though large-scale vaccination programs have mitigated the death toll and hospitalizations, seasonality of spread and new virus variants continue to cause new ‘waves’ of increased infection. As a result, COVID-19 still puts significant strain on healthcare systems worldwide. Even though the pandemic has been put into relative control in many countries, recent examples of virus resurfacing, e.g. the 2022 surge in Shanghai, China^2^ (due to mutations, lack of containment measures, and vaccine resistance) suggest the world is still in danger of further ‘waves’. Insights into factors which can predict mortality and morbidity of patients infected with SARS-CoV-2 can aid physicians, health facility managers and policy makers to make better informed decisions, both at present and in future epidemics. Moreover, the pandemic demonstrated the relative unpreparedness of healthcare systems to deal with many infected patients while providing adequate care to them. One aspect of this unpreparedness can be attributed to the lack of robust and appropriate disease models. Through the pandemic, there was a significant effort to develop algorithms and decision-support systems to aid triaging and patient management. While it is still difficult to say which models and AI tools have been useful, most studies relied on either established or newly-designed clinical scores (e.g. the NEWS-2 score^3^, ROX index^4^, ISARIC-4C^5^ score), classic machine learning classification (e.g. Support Vector Machines^6^), or neural networks/Deep Learning for either imaging^7^ or clinical data^8^ to predict patient outcomes. Of the methods utilised to date, clinical scores have shown most promise. Yet perhaps due to the rapid development and testing of methods, the majority of existing studies have shown significant limitations—e.g. lack of independent test dataset^6,8^, overfitting, miscalibration^9^ (especially for imaging-based deep learning models), non-availability of code implementation, lack of explainability, small sample size, or biased data selection^7,9^.

To overcome these limitations, we adapted an unsupervised algorithm, SuStaIn^10^, to be deployed to data from the first wave of the COVID-19 pandemic. SuStaIn has already shown great promise in in tackling several chronic diseases^11–13^, but it can now be used to gain insights and aid management of shorter-term, infectious disease. We used 11 routinely collected clinical measures on admission to hospital to disentangle distinct clusters of patients (called subtypes) and severity stages of the disease within subtypes, both of which were predictive of inpatient hospital outcomes. Predictions from SuStaIn provide insight into both disease subtypes and severity—a nuance which many models miss. It further balances model complexity, to capture biomarker dynamics, and explainability, which positions it as a useful clinical tool for triaging patients based on their SuStaIn subtype and stage. Unlike other predictive scales or deep-learning models, it is now readily deployable to future infectious disease epidemics and the model implementation is available online.

## Methods

### Population

This study analysed data from the National COVID-19 Chest Imaging Database (NCCID), which comprised COVID-19 positive and negative patients^14,15^. All patients in the study were admitted with suspected COVID-19 infection. In patients with a confirmed positive Polymerase Chain Reaction (PCR) SARS-CoV-2 RNA test, NCCID also collected imaging: Computed Tomography (CT) and Chest X-ray (CXR), as well as clinical information, where the imaging was performed during the hospitalisation period and the salient clinical readings were acquired at admission. The study also included a group of patients who were hospitalized but were subsequently found to be negative for COVID-19. They had to have tested negative on repeated PCR for COVID-19 and not have been admitted to hospital in the subsequent month. All data used was collected from patients admitted to hospital in the UK from January 2020 to January 2021. The data was collected from 14 NHS Hospital Trust centres in the UK, comprising 52 hospitals, which submitted a variable number of cases each.

All data was previously gathered as part of the NCCID study and was stored and analysed in accordance with the established study guidelines as outlined in an earlier work describing the dataset^14^*.* Ethical approval was granted by the UK Health Research Authority and the Scottish Public Benefit Privacy Panel (PBPP), and was also reviewed by NHS Information Governance^14^. Processing of pseudonymised patient data for this study was allowed under a nationally issued Notice under Regulation 3(4) of the Health Service Control of Patient Information Regulations 2002 (COPI). This notice required all hospitals and NHS centres to share and process confidential patient information for COVID-19 purposes (protecting public health, providing healthcare services to the public and monitoring and managing the outbreak)^15^. Subject consent for publication was not required as all data was pseudonymised^14^. All data collection, processing and sharing in the NCCID study was done under the rules and conditions outlined in the Notice. Approval for the retrospective analysis of clinically data and imaging data in NCCID was obtained from the local research ethics committees and Leeds East Research Ethics Committee: 20/YH/0120.

### Data preparation

Even though NCCID enrolled many centres in data collection, the significant load imposed by the ongoing COVID-19 pandemic led to many instances of missing data, especially in the clinical readings at admission. As a result, we used a portion of the NCCID dataset, primarily driven by data completeness. A total of 1344 subjects (referred to as case population) were used in the current study, in addition to 137 COVID-19 negative patients who were utilised as controls for the disease progression model (please see “Subtype and stage inference model”). Manual data quality assurance, curation and standardisation was performed on all clinical data.

We selected eleven clinical tests as biomarkers for disease progression modelling: creatinine, urea, C-reactive protein, lymphocyte count, platelet count, white cell count, respiratory rate, temperature, heart rate, systolic and diastolic blood pressure. Several of these measures have been suggested as being prognostically important in previous survival analyses^3,5,16^. The choice of clinical tests to include in our model was driven by previous use in research and by practicality. All clinical test results were recorded on admission of the patients to hospital.

The 1344 covid-positive cases were split randomly into a training and validation sample of 672 subjects after matching the two populations for age. All model training and tuning was performed solely on the training population and the patients in the validation population were used only at testing.

NCCID data was accessed through a UCL-owned XNAT instance. The Microsoft Azure platform and tools from Microsoft Project InnerEye Open Source Software were used for cloud-based modelling and analysis (https://aka.ms/InnerEyeOSS).

### Subtype and stage inference model

Subtype and Stage Inference (SuStaIn) is an unsupervised learning algorithm that simultaneously identifies clusters (subtypes) and progression sequences (stages) of disease based on worsening biomarker readings. SuStaIn was first developed to model long-term chronic diseases such as Alzheimer’s^10^ and Chronic Obstructive Pulmonary Disease (COPD)^11^. Uniquely, it extracts a temporal (or pseudo-temporal) evolution of disease from single-timepoint, cross-sectional data to account for the inherent progression of diseases. The present study is the first to apply SuStaIn to an infectious disease in its acute phase.

Linear z-score SuStaIn was the chosen SuStaIn model, in which each of the eleven clinical biomarkers was transformed to a z-score with reference to a control population. The control population for this study consisted of 137 patients who were suffering from acute disease (initially suspected to be COVID-19) and were hospitalised but were later determined to not have COVID-19. This population was favourable for usage as controls to SuStaIn since all patients were unwell enough to be admitted to hospital but were not infected with COVID-19. By z-scoring the 11 biomarkers to this population, the effects of COVID-19 infection on the biomarkers were separated from the effects of other acute disease.

Several data preparation steps were carried out prior to initiating modelling with SuStaIn to isolate the COVID-19 signal from other potential covariates. First, the effects of age and sex on all 11 biomarkers were learned in the control population and regressed out from the entire population. Second, the distributions of biomarkers were checked for normality through the Shapiro–Wilk and D’Agostino’s K^2^ test. If a biomarker distribution failed any of the normality tests, a power transform (either the Box-Cox or Yeo-Johnson) was used to improve the normalisation of its distribution. The transformations were applied both on the control and case populations and were necessary since normal distributions are assumed by the linear z-score SuStaIn model.

Finally, each biomarker was transformed into a z-score with reference to the control population, as described earlier. Since some biomarkers were expected to increase or decrease with disease progression, those found to decrease in the case population with reference to the control population (implying negative z-scores), were inverted to ensure all biomarker progression was represented by monotonically increasing z-scores.

Several hyperparameters—model parameters which are not automatically learned, but are instead chosen and optimised by the researcher, were selected—namely the z-score thresholds which represent a stage of progression and the maximum number of subtypes (clusters) to search for. These were tuned and the best-fitting model selected. Table 1 outlines the z-score thresholds selected for each biomarker. When a biomarker reaches a certain z score value (e.g. z = 1 or z = 2), this represented a new disease severity stage.

**Table 1:** The clinical measures (biomarkers) used for SuStaIn modelling.

Biomarker	Unit	Included Z-score thresholds	
		*z* = 1	*z* = 2
Creatinine	μmol/L	x	
Urea	mmol/L	x	
Respiratory rate	breaths/min	x	x
C-reactive protein	mg/L	x	x
Temperature	°C	x	
Systolic BP	mmHg	x	
Diastolic BP	mmHg	x	
Heart rate	beats/min	x	x
Lymphocyte count	10^9^/L	x	x
Platelet count	10^9^/L	x	
White cell count	10^9^/L	x	

Biomarkers were thresholded at certain z-score values to represent a SuStaIn disease severity stage—either when a biomarker reaches a z-score of 1 or a z-score of 2. Each threshold for each clinical measure is marked with an ‘x’ in the table below.

After the model was trained (on the training population), each subject was assigned a SuStaIn subtype and stage. Subtype was assigned by selecting the most probable cluster. Instead of assigning a simple integer stage to each subject, a weighted stage was designated. For each subject, each stage was weighed by the probability of the subject belonging to that stage and the result was then summed, producing a continuous weighted stage. Subjects in the validation population were subtyped and staged using the model trained on the training population.

### Frailty Cox proportional hazards models

To model the survival of patients admitted with COVID-19 infection, the Cox Proportional Hazards (PH) model was used. We used 5 predictor variables in the model: age, sex, subtype, weighted stage, and the subtype-weighted stage interaction. Two outcomes were predicted—time to in-hospital death and time to escalation of patient management. Escalation was defined as in-hospital deterioration which resulted in either ITU admission, intubation or death. The earliest of these 3 events was used as the measure of time to escalation for each patient. Observations were right censored to 6 months after hospital admission as this was the maximum hospital stay for some patients (before discharge or death). To account for the significant variability between centres, a frailty Cox PH model^17^ was adopted with NHS centre as the frailty variable, modelling the random effects in the population.

## Results

### Covid subtypes and severity progression

**Figure 1:**
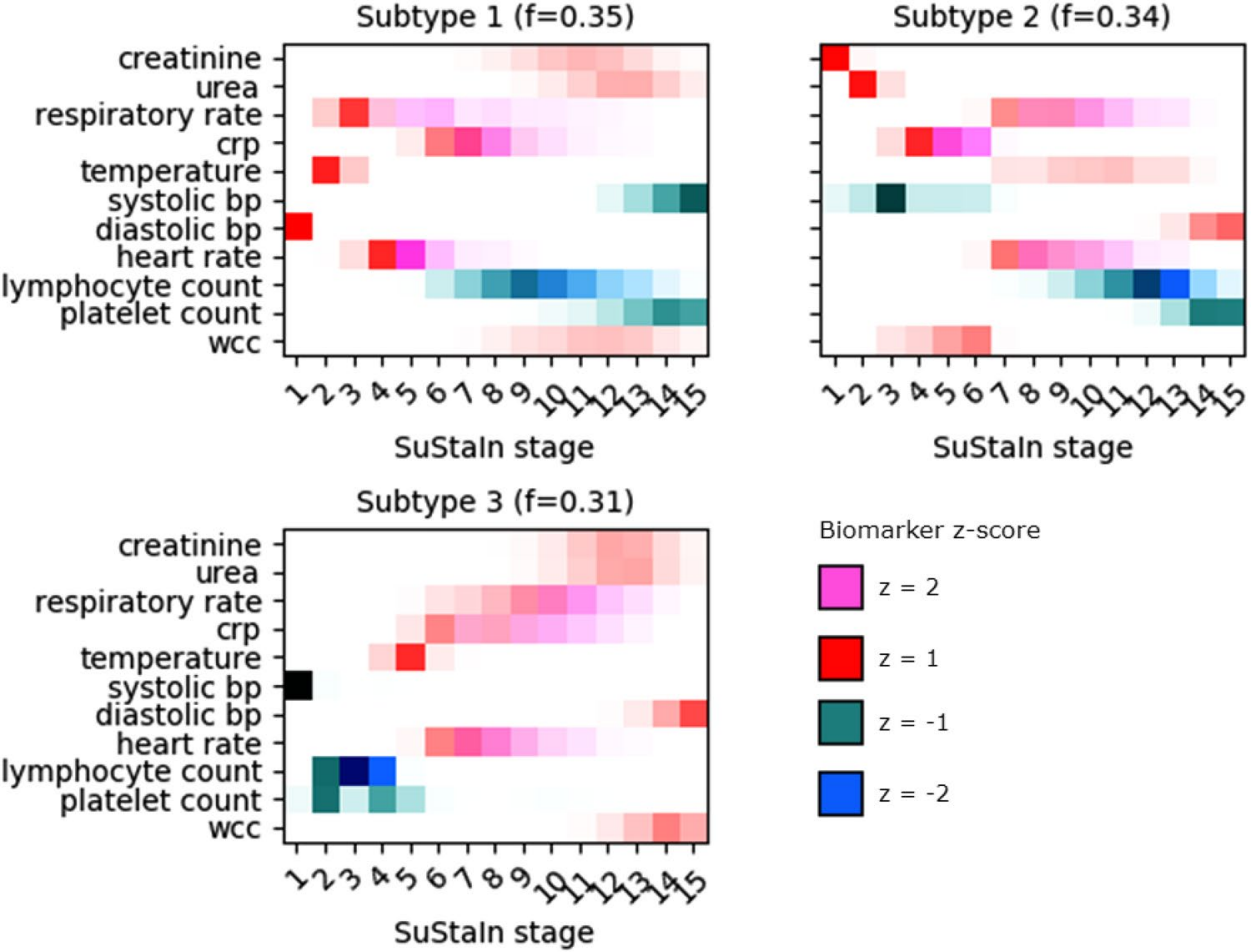
COVID-19 subtypes and disease severity progression. The warm colours represent disease stages progressing towards positive z-scores (z = 1, z = 2) and the cold colours—towards negative z-scores (z = − 1, z = − 2). Increased colour transparency signifies greater uncertainty. The f-value next to each subtype represents the fraction of the training population which was classified as belonging to this subtype.

SuStaIn discovered 3 clinical subtypes of COVID-19 (based on the training population), characterised by distinct in-hospital disease progression. SuStaIn has previously been used to model long-term disease like Alzheimer’s or COPD, which span years, but we adapted it for the relatively short time span of an infectious disease (in-hospital monitoring for up to 6 months). Hence, the disease stages can be interpreted as sequences of progression in the severity of disease within each subtype. We named the three subtypes ‘General Haemodynamic’, ‘Renal’ and ‘Immunological’ (Fig. 1).

#### Subtype 1: general haemodynamic

In this subtype, less severe disease was characterised by high diastolic blood pressure, temperature, respiratory and heart rate, which was then followed by further heart rate increases, elevated CRP and a decrease in lymphocyte levels.

#### Subtype 2: renal

The Renal subtype was characterised by early elevations in creatinine and urea levels, followed by a decrease of systolic blood pressure and an increase in CRP. Unlike the other 2 subtypes, which only exhibit abnormal creatinine and urea in late-stage disease (SuStaIn severity stages 12+), patients with the Renal subtype experienced these abnormalities early in their disease severity progression.

#### Subtype 3: immunological

In the Immunological subtype, COVID-19 began with abnormally low systolic blood pressure, followed by a cascade of decreases in lymphocyte and platelet count and then elevated temperature, heart rate and CRP levels at more advanced disease.

In all subtypes, abnormalities in the systolic and diastolic blood pressures seemed to be separated—being placed at the opposite ends of SuStaIn stage in all three subtypes.

### Data exploration

SuStaIn modelling revealed a large proportion of patients were assigned to SuStaIn stage 0—a disease state, which was very similar to the control population. These patients were grouped into a separate, Normal-appearing Subtype 0—290 patients from the training population and 317 patients from the validation population were found to belong to this subtype. These subjects had a milder COVID-19 presentation and were later found to have a much higher probability of survival.

Furthermore, for the following biomarkers, progression represented a decrease rather than an increase in the real-value biomarker readings: systolic blood pressure, lymphocyte count and platelet count. This meant that for these 3 biomarkers, the average biomarker readings were lower in the case population as compared to the control population. Advancing of SuStaIn stages for these 3 biomarkers, therefore, represented decreases in their absolute values. For clinical context, Table 2 presents an overview of the absolute values of each biomarker for each subtype. General demographic data for the training and validation populations, in aggregate, and also split by subtype, can be found in Table 3.

**Table 2:** Descriptive statistics for the 11 biomarkers in the entire case population, split by subtype.

Biomarker	Creatinine (μmol/L)			Urea (mmol/L)			Respiratory rate (breaths/min)		
	Mean	Std	Median	Mean	Std	Median	Mean	Std	Median
Subtype 0	82.8	30.9	76.0^a^	5.9	2.8	5.3^a^	20.1	3.9	20.0^a^
Subtype 1	104.7	99.1	87.0^a^	7.9	5.4	6.4^b^	28.2	7.6	28.0^b^
Subtype 2	228.7	211.7	156.5^b^	15.8	8.9	13.8^c^	23.4	7.5	21.0^c^
Subtype 3	102.9	80.2	86.0^a^	8.3	6.0	7.1^b^	22.8	5.7	21.0^c^

	Temperature (°C)			Systolic BP (mmHg)			Diastolic BP (mmHg)		
	Mean	Std	Median	Mean	Std	Median	Mean	Std	Median
Subtype 0	37.0	0.9	36.9^a^	134.3	23.2	131.0^a^	75.1	12.6	75.0^a^
Subtype 1	38.1	1.0	38.2^b^	148.9	23.3	147.0^b^	88.9	16.7	87.0^b^
Subtype 2	37.2	1.1	37.1^a^	120.9	20.4	120.0^c^	68.3	12.6	69.0^c^
Subtype 3	37.6	1.1	37.7^d^	118.4	20.2	118.0^c^	68.2	12.3	68.0^c^

	Lymphocyte count (10^9^/L)			Platelet count (10^9^/L)			WCC count (10^9^/L)		
	Mean	Std	Median	Mean	Std	Median	Mean	Std	Median
Subtype 0	1.4	2.0	1.1^a^	242.7	104.2	225.0^a^	7.4	3.7	6.7^a^
Subtype 1	1.0	0.7	0.8^b^	249.8	173.4	223.0^a^	8.7	4.1	7.8^b^
Subtype 2	1.3	1.9	0.9^a,b^	256.7	128.7	233.5^a^	12.1	6.8	10.8^c^
Subtype 3	0.5	0.2	0.4^c^	159.4	76.7	158.0^b^	6.9	5.5	5.8^a^

	CRP (mg/L)			Heart rate (beats/min)					
	Mean	Std	Median	Mean	Std	Median			
Subtype 0	62.3	64.2	40.2^a^	84.7	16.1	84.0^a^			
Subtype 1	116.7	117.6	90.0^b^	105.8	19.9	104.0^b^			
Subtype 2	165.2	107.0	148.1^c^	91.3	19.3	91.0^c^			
Subtype 3	105.6	76.8	89.5^b^	92.4	19.3	90.5^c^			

Subtype 0 represents the 'normal' looking subtype, which is most similar to the control population. Std—standard deviation. One-way ANOVA with the Tukey post-hoc tests performed between subtypes for each biomarker: results indicated with labels (a, b, c, d)—subtypes with a significant pairwise difference have different labels, while subtypes which were not significantly different share the same labels.

**Table 3:** Demographics per population and subtype.

	Training					Validation				
	All	Subtype 0	Subtype 1	Subtype 2	Subtype 3	All	Subtype 0	Subtype 1	Subtype 2	Subtype 3
Age [mean (std)]	70.0 (16.2)	67.0 (16.9)	69.2 (15.0)	74.3 (15.5)	73.0 (15.1)	69.5 (16.2)	66.2 (17.1)	71.8 (14.9)	73.9 (14.4)	71.4 (14.7)
Sex [% female)	59.8	55.2	63.4	60.6	67.0	61.6	59.3	68.2	63.0	59.8
Smoking status [% in each category]	N: 32.7 E: 20.0 C: 3.4 U: 43.4	N:32.8 E: 20.4 C: 3.2 U: 43.6	N: 36.1 E: 19.4 C: 3.7 U: 40.7	N: 30.5 E: 16.1 C: 2.6 U: 50.8	N: 31.3 E: 27.7 C: 4.8 U: 36.1	N: 31.7 E: 20.2 C: 4.6 U: 43.4	N: 31.7 E: 18.3 C: 4.2 U: 45.8	N: 31.1 E: 23.7 C: 6.5 U: 38.7	N: 32.5 E: 20.2 C: 3.5 U: 43.9	N: 31.9 E: 22.0 C: 5.5 U: 40.7
Mortality [% died]	32.9	18.6	38.2	52.8	38.5	29.9	18.9	32.7	47.1	37.4
Days to death [mean (std)]	125.5 (78.5)	149.3 (64.4)	115.4 (82.8)	93.3 (83.7)	116.1 (81.5)	130.3 (76.8)	149.1 (64.6)	124.4 (80.4)	101.5 (85.1)	117.6 (81.4)
Escalation [% escalated]	41.1	22.4	58	57.7	48.6	37.6	25.2	45.5	55.8	43.0
Days to escalation [mean (std)]	110.5 (83.9)	142.4 (70.2)	78.5 (86.8)	84.4 (83.7)	98.1 (85.0)	115.5 (83.4)	137.4 (73.9)	101.2 (87.0)	83.4 (86.6)	107.1 (84.7)

Smoking status: N—never, E—ex-smoker, C—current smoker, U—unknown. No significant differences were found in any variable between the training and validation populations (using t-tests for continuous variables and chi-squared tests for nominal and binary variables).

### Cox proportional hazards (PH) frailty model

SuStaIn subtype and weighted stage was found to be a significant predictor of both in-hospital escalation of patient management and in-hospital mortality for patients admitted with COVID-19. Cox PH models were fitted separately on the training and test populations and then set against one another to confirm consistency of the results. The Kaplan–Meier curves and model coefficients were examined as a form of validation, as suggested previously^18^.

#### Predicting escalation of patient management using SuStaIn

Table 4 is a summary of the multivariable Cox proportional hazards models fitted to both the training and validation population, with a frailty term accounting for bias between submitting NHS Hospital trusts. The results were consistent between populations, suggesting that SuStaIn subtype and stage generalise as predictors of escalation between 2 randomly selected populations (albeit in patients whose data was collected as part of the same study). The interaction of subtype and weighted stage, moreover, produced the greatest overlap in coefficients.

**Table 4:** Multivariable Cox Proportional Hazards modelling of Time to Escalation in the training and validation population.

Covariate	Training				Validation			
	HR	Lower 95%	Upper 95%	p-value	HR	Lower 95%	Upper 95%	p-value
Age	1.01	1.00	1.02	0.01	1.01	1.00	1.01	0.29
Sex	0.80	0.62	1.03	0.08	0.59	0.45	0.78	0.00
Subtype 1	4.05	2.08	7.88	0.00	2.69	1.32	5.47	0.01
Subtype 2	3.73	1.85	7.49	0.00	2.77	1.41	5.45	0.00
Subtype 3	5.04	2.37	10.74	0.00	2.58	1.28	5.17	0.01
Weighted stage	2.74	1.35	5.56	0.01	2.99	1.58	5.66	0.00
Subtype 1: wstage	0.44	0.22	0.90	0.02	0.39	0.20	0.75	0.00
Subtype 2: wstage	0.42	0.21	0.86	0.02	0.42	0.22	0.80	0.01
Subtype 3: wstage	0.35	0.17	0.72	0.00	0.38	0.20	0.73	0.00

The hazard ratios, HR, (and consequently the exponent of model coefficients) between the training and validation populations show significant overlap. The effects of the frailty variable—NHS Hospital trust, are not shown as there are 14 centres in the population.

wstage: weighted SuStaIn stage; sex 0: female; sex 1: male; variable interactions denoted with ‘:’

Model concordance was good and was nearly equal in the Cox models fitted to both the training (C index of 0.69, 95% CI 0.66–0.72) and validation (C index of 0.69, 95% CI 0.65–0.72) populations.

**Figure 2:**
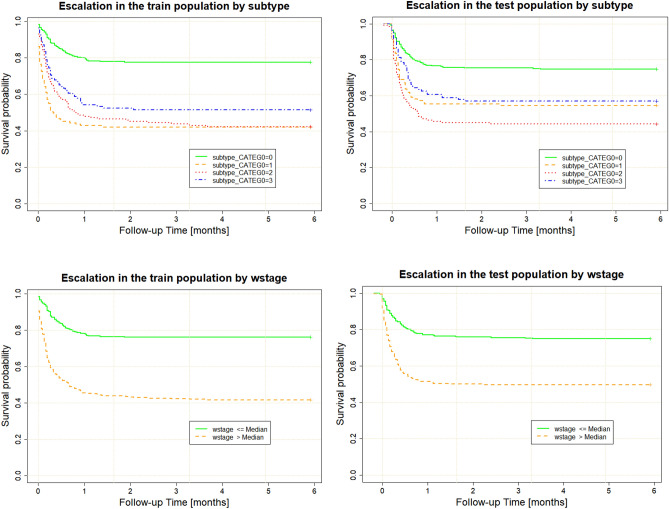
Kaplan–Meier plots for 6-month in-hospital escalation of treatment for the training (left) and validation (right) population. wstage—weighted SuStaIn stage.

Early SuStaIn stages and Subtype 0 were found to predict much less frequent in-hospital escalation of treatment as compared to the other 3 subtypes (Fig. 2). Among the three subtypes, patients assigned to the Immunological subtype (subtype 3) were least likely to experience escalation of treatment, while the General Haemodynamic (subtype 1) and Renal (subtype 2) subtypes were more likely to require treatment escalation while hospitalised (Fig. 2). The Kaplan–Meier curves for SuStaIn subtypes were generally consistent in the training and validation populations. The only subtype showing poorer calibration between populations was the haemodynamic subtype where the KM curves differed between populations.

**Figure 3:**
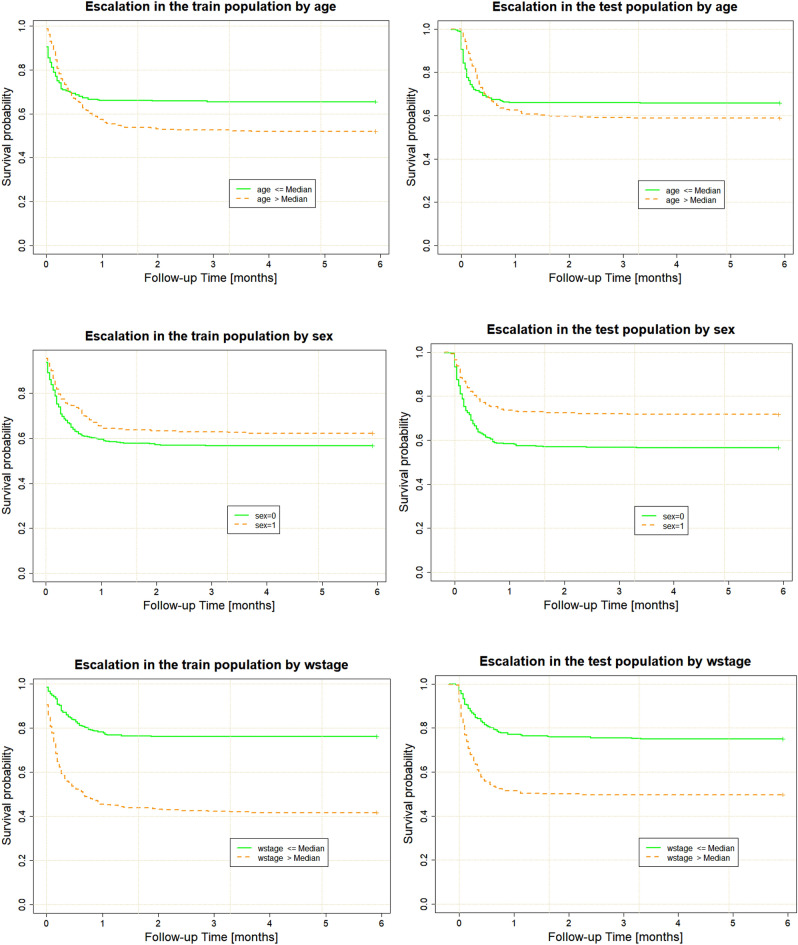
SuStaIn stage provides better discrimination of time to escalation than age or sex: left—training population, right—validation population. wstage—weighted SuStaIn stage. sex 0—female, sex 1—male.

SuStaIn stage on its own had significant discrimination for the need for escalation of treatment (Fig. 3) and was a better predictor of escalation than patient age or sex.

#### Mortality prediction using SuStaIn

SuStaIn subtype and stage were also good predictors of in-hospital mortality. As shown in Table 5, the hazard ratio confidence intervals show good overlap between training and validation populations. For determining mortality, subtype and weighted stage on their own were better predictors than the subtype–stage interaction (which did not achieve significance at the 0.05 threshold in the training population). Model concordance for both the training and validation populations was equal: C index of 0.74, 95% CI 0.71–0.77 on the training population and C index of 0.74, 95% CI 0.71–0.77 on the validation population, showing a slightly better concordance than the models for escalation of patient management.

**Table 5:** Multivariable Cox proportional hazards analyses modelling time to death in the training and validation groups.

Covariate	Training				Validation			
	HR	Lower 95%	Upper 95%	p-value	HR	Lower 95%	Upper 95%	p-value
Age	1.04	1.03	1.05	0.00	1.04	1.03	1.05	0.00
Sex	0.85	0.65	1.13	0.26	0.69	0.51	0.94	0.02
Subtype 1	2.35	1.07	5.14	0.03	2.35	1.00	5.51	0.05
Subtype 2	3.39	1.58	7.26	0.00	2.28	1.04	5.03	0.04
Subtype 3	3.07	1.30	7.25	0.01	2.55	1.14	5.68	0.02
Weighted stage	2.32	1.01	5.30	0.05	2.72	1.28	5.78	0.01
Subtype 1: wstage	0.52	0.22	1.19	0.12	0.42	0.19	0.90	0.03
Subtype 2: wstage	0.49	0.21	1.12	0.09	0.47	0.22	1.01	0.05
Subtype 3: wstage	0.44	0.19	1.02	0.06	0.43	0.20	0.92	0.03

HR: hazard ratio; wstage: weighted SuStaIn stage; sex 0: female; sex 1: male.

SuStaIn subtype 0 was, as with the models of treatment escalation, characterised by significantly lower in-hospital mortality. The Renal subtype demonstrated the highest risk of dying in hospital, showing consistent results of ~ 50% survival at 6 months in both the training and validation populations. Subtypes 1 and 3 had very similar prognoses in the training population (at ~ 70% 6-month survival), but subtype 3 showed slightly worse calibration in the validation population and a slightly worse survival.

**Figure 4:**
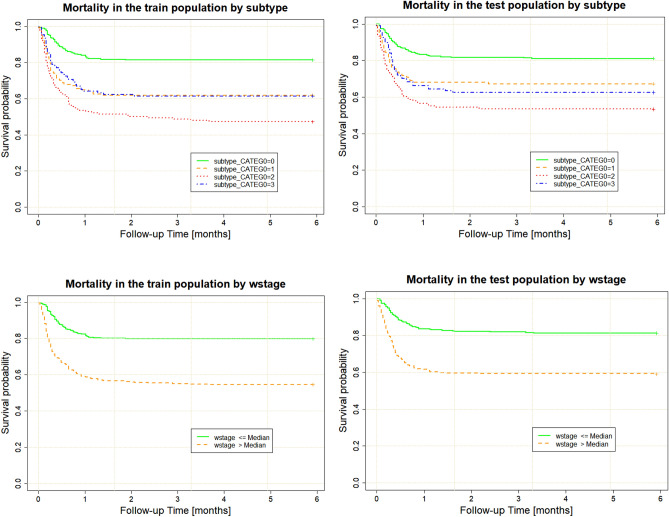
Kaplan–Meier plots for 6-month in-hospital mortality for the training (left) and validation (right) population. Wstage—weighted SuStaIn stage.

SuStaIn stage was also, independently, associated with higher risk of in-hospital mortality (Fig. 4).

**Figure 5:**
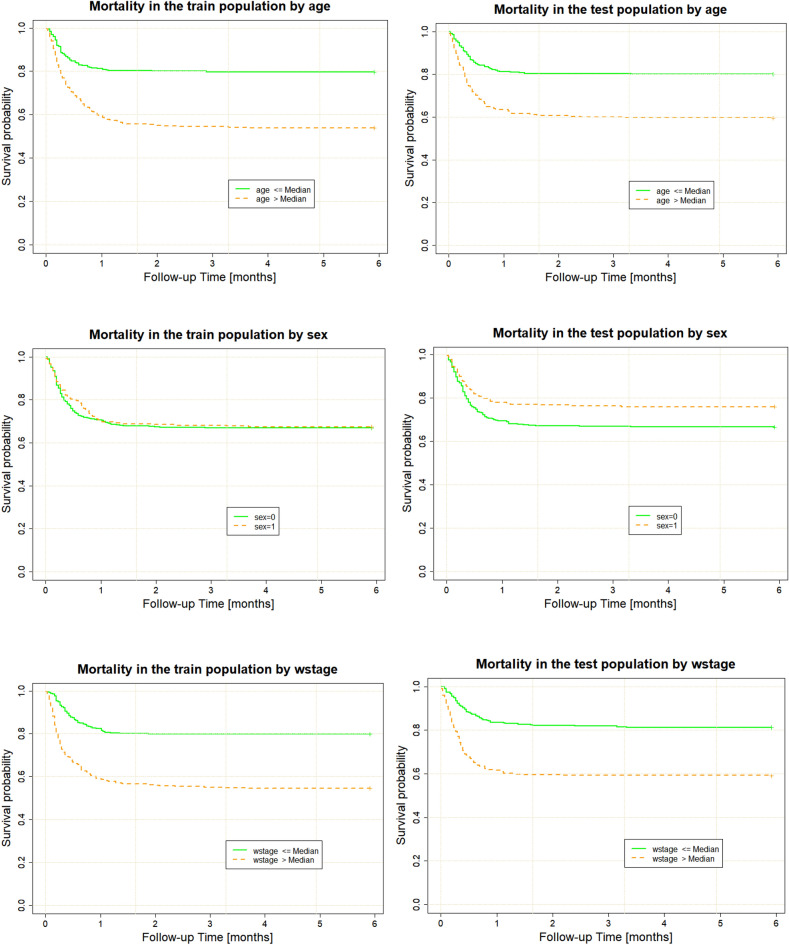
SuStaIn stage provides better discrimination for 6-month in-hospital mortality than age or sex (left—training population, right—validation population. wstage—weighted SuStaIn stage. sex 0—female, sex 1—male.

As expected, age was a strong predictor of in-hospital mortality, with older patients being at higher risk. Sex had a smaller effect on mortality, but calibration for sex was poor (Fig. 5), probably as a consequence of the random sampling used when creating the training and validation populations, which led to a slightly different proportion of men and women (Table 3).

## Discussion

We demonstrated that an unsupervised machine learning model, traditionally used for long-term disease progression modelling—SuStaIn, is readily adaptable to a pandemic of viral disease. The three SuStaIn subtypes we discovered likely represent disease involvement in distinct organ systems while SuStaIn stages provide the required gradation to disease severity in patients with COVID-19, which is valuable for risk stratification and outcome prediction. The zeroth subtype also represents a valuable signal, characterizing patients who have been admitted to hospital but were in fact at low risk of death or escalation of treatment. The robustness of our results further highlights our model’s significance as a readily available clinical tool in future epidemics of influenza or further COVID-19 variants.

Several studies have previously investigated factors associated with differing severity of COVID-19 infection on a number of large-scale datasets, such as the NCCID, ISARIC, PHOSP-COVID^19^. As a result, various clinical measures and biomarkers have been derived for use as prognostic factors for patients diagnosed with COVID-19. Patients admitted for COVID-19 have been reported to have a ~ 5 times higher hazard ratio for death, ~ 4 higher hazard ratio for mechanical ventilation and 2.41 higher hazard ratio for being admitted to an intensive care unit (ITU)^20^ compared to influenza. In addition to the pulmonary manifestations of pneumonia and ARDS^21^, COVID-19 infection is further associated with injuries to other organs including: acute kidney injury, deep venous thrombosis, stroke, sepsis and sudden cardiac death^20^. To predict short-to-medium term outcomes (in-hospital death or ITU admission), the National Early Warning Score (NEWS2)—an existing risk stratification tool was initially used. However, studies have shown its low discrimination power when applied to COVID-19 patients^3,4^. A combination of NEWS2 with 8 further routinely collected blood and clinical measures (supplemental oxygen flow rate, urea, age, oxygen saturation, C-reactive protein, estimated glomerular filtration rate, neutrophil count, neutrophil/lymphocyte ratio) improved its discrimination power for severe COVID-19 outcomes, but model calibration remained poor^3^, necessitating the development of COVID-19 specific patient stratification and prognostication tools. One such tool was the ROX index, evaluated by Prower et al.^4^ The ROX index represents the ratio between the peripheral oxygen saturation (SpO2), and the concentration of oxygen in inhaled oxygen (21% in room air), divided by the patient’s respiratory rate and was developed to indicate the need for intubating patients suffering from hypoxia. The authors found that the ROX index predicted adverse events 5 h earlier than NEWS2 and provided a clinically useful warning signal. The study emphasized the prognostic importance associated with a deterioration in respiratory parameters in escalation management of COVID-19. Investigation into other prognostic factors for COVID-19 in hospitalized patients included the development of the ISARIC 4C Mortality Score^5^. The score ranges from 0 to 21 points and included eight routinely collected clinical readings: age, sex, number of comorbidities, respiratory rate, peripheral oxygen saturation, level of consciousness, urea level, and C reactive protein^5^. The ISARIC 4C Mortality score was developed on a large UK population (~ 58,000 patients), as part of the ISARIC study^22^ and the authors reported excellent discrimination of the score for in-hospital mortality and, more importantly, very good model calibration suggesting applicability of the score when used in new centres and populations. The performance of the score in predicting mortality was also superior and the authors compared their score to 15 other risk stratification scores^5^. The ISARIC 4C consortium further developed a Deterioration model (based on multiple logistic regression) to predict not only mortality, but clinical deterioration, defined as admission to ITU or need for mechanical ventilation^16^. The model displayed convincing discrimination and calibration by using 11 clinical biomarkers: age, sex, respiratory rate, oxygen saturation, room air or oxygen, level of consciousness (Glasgow Coma Scale), nosocomial infection, radiographic infiltrates, urea concentration, lymphocyte count and C reactive protein^16^.

While it is difficult to make direct model comparison due to an only partial overlap in the used clinical measures/biomarkers, we demonstrated that by using a purely cross-sectional clinical and biological data at admission for COVID-19 (11 routinely collected biomarkers) and modelling disease severity progression with SuStaIn, clinically meaningful subtypes and stages of COVID-19 can be derived. This departs from the idea of a one-size-fits-all index and allows us to model involvement in different organ systems through SuStaIn subtypes. In addition to being predictive of in-hospital outcomes, our results can be valuable for organ-specific studies of damage from COVID-19. Previous studies, using tools such as the ISARIC 4C^22^ or ROX index^4^ tried to use a single scale to predict patients outcomes and prioritise treatment. However, this view, while it has shown clinical utility, may miss the inherent nuance in the progression patterns of patients infected with Sars-CoV-2. In terms of triaging, our model can be used to assign patients admitted to hospital for COVID-19 to one of the 4 subtypes by simply taking the readings of the 11 biomarkers we used. Subtype 0 patients, while ill enough to be hospitalised, can be classified as ‘low-risk’ for either experiencing escalation of treatment or dying in hospital. Subtype 3 patients, similarly, are at a lower risk, but patients assigned to Subtypes 1 or 2, and especially at their more advanced SuStaIn stages, should be prioritised for treatment and monitored more closely.

The disease subtypes discovered by SuStaIn modelling broadly affect different systems within the body and consequences from COVID-19 in these systems have been previously described. The Renal subtype (Subtype 2) is consistent with several studies which identified some COVID-19 patients experiencing significant kidney problems or even acute kidney injury (AKI)^23,24^. In the consensus report, patients suffering AKI were at significantly increased risk of all-cause death in hospital^23^. Our model further provides stages within this subtype which can differentiate patients by considering all 11 readings. While patients admitted with just elevated urea and creatinine, for example, might belong to subtype 2, if they are relatively normal in the other 9 biomarkers, they may be assigned to an early SuStaIn stage. A clinician might then monitor development of further changes in biomarkers to diagnose severity progression within the Renal subtype, which can inform risk determination and treatment.

The General Haemodynamic subtype (Subtype 1) can be hypothesised to relate to the common blood-clotting and hyper-inflammatory effects, described in a number of studies^25,26^. An interesting finding which our model uncovered is that late-stage disease patients who are at the greatest risk of escalation and dying within this subtype (advanced SuStaIn stage) experience a drop in their lymphocytes, platelets, and systolic blood pressure. An early decrease in platelet count was found to predict mortality in a study in Wuhan^27^, which might represent a possible depletion of systemic platelets due to significant clotting in the lung. Another study also reported a trend of rather sharply dropping platelets in non-survivors over multiple timepoints during hospitalisation^28^. Indeed, late SuStaIn stages in both Subtypes 1 and 2 were characterised by a drop in platelet count—those were the patients at greatest risk of dying in hospital. Although our work reconstructs disease severity progression from just a single timepoint reading, patients assigned to the later SuStaIn stages of Subtypes 1 and 2 might have already had a reduced platelet count by the time of hospital admission (effectively more advanced disease). By examining the absolute values of platelet counts for these patients, the same ranges of values (between 100 and 150 × 10^9^/L) were discovered in late-stage patients in our study and in Yang et al.^28^ The decreases in total lymphocyte count, characteristic of the late SuStaIn stages in subtype 1 and 2 patients is also consistent with a meta-analysis of 20 studies, which determined this decrease to be closely associated with advanced severity of disease^29^.

The Immunological subtype (subtype 3), on the other hand showed lower levels of lymphocytes and platelets in the lowest-risk, early disease stages. These findings highlight the importance of signals contained within the multitude of biomarkers routinely collected during medical care. Our model aggregated several of these biomarkers and benefited from the inferred clustering of disease and stages of disease severity rather than employing a one-size-fits-all approach for triaging and prognostication. While decreased lymphocytes and platelets might imply a high risk of death and escalation of treatment when occurring after a series of haemodynamic (Subtype 1) or renal (Subtype 2) symptoms, they might indicate lower risk if occurring without these symptoms as seen in Subtype 3. SuStaIn’s ability to disentangle sequences of progressing severity and subtype simultaneously provides a far more detailed picture than a single score for all patients.

Our approach also identified an interesting dissociation of systolic and diastolic blood pressure in all subtypes. Namely, the abnormally increased diastolic blood pressure and abnormally decreased systolic blood pressure were always placed at opposite ends of disease severity stages. This suggests that instead of one of the blood pressure phases indicating severe disease, it might be the effectively decreased pressure range between systole and diastole (pulse pressure) which hallmarked advanced COVID-19 and increased a patient’s chance of both escalation of treatment and death. This signal merits further investigation as two studies indicated that a high variability of blood pressure in COVID-19 patients is associated with poorer outcomes^30^ and, interestingly, that patients who have recovered from COVID-19 tend to have impaired aortic distensibility^31^.

The main strength of the present work is that it was able to demonstrate clinically significant differences in both escalation of treatment and mortality for patients hospitalised for COVID-19, based on 11 routine and easy to collect clinical measurements. We discovered 3 distinct subtypes of COVID-19, which might imply different underlying pathophysiology and disease course in different patients. Although the data we used was collected as part of a single study (the NHSX NCCID), it came from hospitals and NHS trusts throughout the UK and included patients from diverse socio-economic and racial backgrounds. We further employed one of the most challenging techniques for the validation of our Cox Proportional Hazards models—replication on a separate sample of patients. Our model can be readily applied, tested, and tuned on a larger sample of patients (e.g., from different studies) using the 11 biomarkers we studied. More broadly, our model can be further augmented should a more complete set of biomarkers, or other feasible biomarkers become available.

There were several limitations to this study. Methodologically, SuStaIn was developed for modelling long-term, chronic disease. This was the first time it was adapted to severe infectious disease. One of its assumptions is that biomarkers can only become more abnormal with time. This means that it cannot inherently derive the transient drops and increases in biomarkers, which might happen while a patient is hospitalised. Nevertheless, the model is still appropriate for stratification of patients and triaging since it focuses on the severe period of disease when patients are hospitalised and deteriorating. All clinical measurements in the NCCID were performed in this period. Hence, in this sense the learned model represents a progression of severity of disease and does not currently capture recovery. Furthermore, while the learned disease severity progression is currently unidirectional, the model poses no constraints on staging patients to an earlier (less severe) stage in case data was available for a follow-up visit. Hence, at the individual patient level, recovery can be modelled. Future work on making SuStaIn even more useful for shorter term infectious disease outbreaks could also relax the assumption of unidirectionality in disease progression, to capture potential population-wide increases and declines in health.

The data which was available for this study also had several limitations. First, the NCCID dataset did not track the presence of coronavirus variants (Alpha, Beta, Gamma, Delta, Omicron)^15,32^ and this information would have been useful for disease modelling since the population likely included different virus variants. However, the common nature of the biomarkers used in our models opens the way for relatively easy validation when new data becomes available. A follow-up timepoint to validate disease progression, as well as availability of additional variables such as patient blood type would also have benefitted our study. Furthermore, there was a risk of false negative PCR tests across the population, which might have caused presence of COVID-19 positive patients in the control population. Finally, the specific causes of death, for example cardiac arrest or pulmonary embolism due to COVID-19, were not recorded in the study—the availability of these would have brought further insight into the pathophysiology of COVID-19.

In conclusion, we found that by using 11 common clinical readings at admission to hospital for COVID-19, we could learn distinct COVID-19 subtypes and disease severity stages, which are predictive of patient outcomes. Importantly, we’ve adapted SuStaIn for use in further infectious disease flares and the model can be readily tune or retrained to capture a finer-grained picture of disease, which can aid patient triaging and resource prioritisation.

## Data Availability

The current study analysed data which was previously collected as part of the NCCID^15^. As described in the dataset overview study^14^, all data from the NCCID is available to any user by submitting an application through a rigorous Data Access Request (DAR) and then following the described procedure outlined in https://nhsx.github.io/covid-chest-imaging-database.
