# Author Correction: Delineating COVID-19 subgroups using routine clinical data identifies distinct in-hospital outcomes

**DOI:** 10.1038/s41598-023-45343-5

**Published:** 2023-10-26

**Authors:** Bojidar Rangelov, Alexandra Young, Watjana Lilaonitkul, Shahab Aslani, Paul Taylor, Eyjólfur Guðmundsson, Qianye Yang, Yipeng Hu, John R. Hurst, David J. Hawkes, Joseph Jacob, Тhe NCCID Collaborative, Pardeep Bains, Dominic Cushnan, Mark Halling-Brown, Joseph Jacob, Emily Jefferson, Francois Lemarchand, Anastasios Sarellas, Daniel Schofield, James Sutherland, Mathew Watt, Daniel Alexander, Hena Aziz, John R. Hurst, Emma Lewis, Gerald Lip, Peter Manser, Philip Quinlan, Neil Sebire, Andrew Swift, Smita Shetty, Peter Williams, Oscar Bennett, Samie Dorgham, Alberto Favaro, Samantha Gan, Tara Ganepola, Gergely Imreh, Neha Puri, Jonathan Carl Luis Rodrigues, Helen Oliver, Benjamin Hudson, Graham Robinson, Richard Wood, Annette Moreton, Katy Lomas, Nigel Marchbank, Chinnoi Law, Harmeet Chana, Nemi Gandy, Ban Sharif, Leila Ismail, Jaymini Patel, Debbie Wai, Liz Mathers, Rachel Clark, Anisha Harrar, Alison Bettany, Kieran Foley, Carla Pothecary, Stephen Buckle, Lisa Roche, Aarti Shah, Fiona Kirkham, Hannah Bown, Simon Seal, Hayley Connoley, Jenna Tugwell-Allsup, Bethan Wyn Owen, Mary Jones, Andrew Moth, Jordan Colman, Giles Maskell, Daniel Kim, Alexander Sanchez-Cabello, Hannah Lewis, Matthew Thorley, Ross Kruger, Madalina Chifu, Nicholas Ashley, Susanne Spas, Angela Bates, Peter Halson, Chris Heafey, Caroline McCann, David McCreavy, Dileep Duvva, Tze Siah, Janet Deane, Emily Pearlman, James MacKay, Melissa Sia, Esme Easter, Doreen Brookes, Paul Burford, Ramona-Rita Barbara, Thomas Payne, Mark Ingram, Bahadar Bhatia, Sarah Yusuf, Fiona Rotherham, Gayle Warren, Angela Heeney, Angela Bowen, Adele Wilson, Zahida Hussain, Joanne Kellett, Rachael Harrison, Janet Watkins, Lisa Patterson, Tom Welsh, Dawn Redwood, Natasha Greig, Lindsay Van Pelt, Susan Palmer, Kate Milne, Joanna Tilley, Melissa Alexander, Amy J. Frary, Judith L. Babar, Timothy Sadler, Edward Neil-Gallacher, Sarah Cardona, Avneet Gill, Nnenna Omeje, Claire Ridgeon, Fergus Gleeson, Annette Johnstone, Russell Frood, Mohammed Atif Rabani, Andrew Scarsbrook, Mark D. Lyttle, Stephen Lyen, Gareth James, Sarah Sheedy, Kiarna Homer, Alison Glover, Ben Gibbison, Jane Blazeby, Mai Baquedano, Thomas Payne, Teresa Jacob, Sisa Grubnic, Tony Crick, Debbie Crawford, Fiona Prestwood, Margaret Cooper, Mark Radon

**Affiliations:** 1 Satsuma Lab, Centre for Medical Image Computing (CMIC), University College London, London, UK; 2 Department of Neuroimaging, King’s College London, London, UK; 3 Institute of Health Informatics, University College London, London, UK; 4 Wellcome/EPSRC Centre for Interventional and Surgical Sciences, University College London, London, UK; 5 Centre for Medical Image Computing, University College London, London, UK; 6 UCL Respiratory, University College London, London, UK; 7 AI Lab, NHSX, Skipton House, 80 London Road, London, SE1 6LH UK; 8 Scientific Computing, Royal Surrey NHS Foundation Trust, Egerton Road, Guildford, GU2 7XX UK; 9 Health Data Research UK, Gibbs Building, 215 Euston Road, London, NW1 2BE UK; 10 Health Informatics Centre (HIC), School of Medicine, University of Dundee, Dundee, DD1 4HN UK; 11 Health Informatics Centre (HIC), School of Medicine, University of Dundee, (Main Level 5 Corridor), Second Floor, Level 7, Mailbox 15, Ninewells Hospital & Medical School, Dundee, DD1 9SY2 UK; 12 Department of Scientific Computing, Royal Surrey NHS Foundation Trust, Egerton Road, Guildford, GU2 7XX England; 13 Centre for Vision, Speech and Signal Processing, University of Surrey, Guildford, England; 14 North East Scotland Breast Screening Programme, NHS Grampian, Aberdeen, UK; 15 Digital Research Service, University of Nottingham, Nottingham, UK; 16 School of Medicine, University of Nottingham, Nottingham, UK; 17 Great Ormond Street Hospital, London, UK; 18 Department of Infection, Immunity and Cardiovascular Disease, University of Sheffield, Sheffield, S10 2RX UK; 19 Faculty, 54 Welbeck Street, London, W1G 9XS UK; 20 Royal United Hospitals Bath NHS Foundation Trust, Bath, UK; 21 Brighton and Sussex University Hospitals NHS Trust, Brighton, UK; 22 London North West University Healthcare NHS Trust, Harrow, UK; 23 George Eliot Hospital NHS Trust, Nuneaton, UK; 24 Cwm Taf Morgannwg University Health Board, Caerphilly, UK; 25 Hampshire Hospitals NHS Foundation Trust, Basingstoke, UK; 26 Betsi Cadwaladr University Health Board, Bangor, UK; 27 Ashford and St Peter’s Hospitals, Ashford, UK; 28 Royal Cornwall Hospitals NHS Trust, Truro, UK; 29 Sheffield Children’s NHS Foundation Trust, Sheffield, UK; 30 Liverpool Heart and Chest Hospital NHS Foundation Trust, Liverpool, UK; 31 Norfolk and Norwich University Hospitals NHS Foundation Trust, Norfolk, UK; 32 Royal Surrey NHS Foundation Trust, Guildford, UK; 33 Sandwell and West Birmingham NHS Trust, Birmingham, UK; 34 West Suffolk NHS Foundation Trust, Bury Saint Edmunds, UK; 35 Somerset NHS Foundation Trust, Taunton, UK; 36 Cambridge University Hospitals NHS Foundation Trust, Cambridge, UK; 37 Imperial College Healthcare NHS Trust, London, UK; 38 Oxford University Hospitals NHS Foundation Trust, Oxford, UK; 39 Leeds Teaching Hospitals NHS Trust, Leeds, UK; 40 University Hospitals Bristol NHS Foundation Trust, Bristol, UK; 41 St George’s Hospital NHS Foundation Trust, London, UK; 42 University Hospitals Of Morecambe Bay NHS Foundation Trust, Kendal, UK; 43 The Walton Centre, Liverpool, England

Correction to: *Scientific Reports* 10.1038/s41598-023-32469-9, published online 20 June 2023

The original version of this Article contained an error in the name of author, Andrew Scarsbrook which was incorrectly given as Prof Andrew Scarsbrook. He is a member of the NCCID Collaborative team.

The original Article has been corrected.

